# From Patterns to Projections: A Spatiotemporal Distribution of Drug-Resistant Tuberculosis in Paraná, Brazil (2012–2023)

**DOI:** 10.3390/pathogens14101046

**Published:** 2025-10-16

**Authors:** Laiz Mangini Cicchelero, Gustavo Cezar Wagner Leandro, Luciano de Andrade, Jean Eduardo Meneguello, Katiany Rizzieri Caleffi-Ferracioli, Rosilene Fressatti Cardoso, Regiane Bertin de Lima Scodro

**Affiliations:** 1 Postgraduate Program in Health Sciences, State University of Maringá (UEM), Maringá 87020-900, Brazil; gustavocezarwl@gmail.com (G.C.W.L.); landrade@uem.br (L.d.A.); jemeneguello@uem.br (J.E.M.); rfcardoso@uem.br (R.F.C.); rblscodro@uem.br (R.B.d.L.S.); 2 Postgraduate Program in Biosciences and Pathophysiology, State University of Maringá (UEM), Maringá 87020-900, Brazil; krcferracioli2@uem.br

**Keywords:** tuberculosis, multidrug-resistant, spatio-temporal analysis, epidemiology, public health surveillance, time factors

## Abstract

Drug-resistant tuberculosis represents a challenge with high potential for spread. This ecological study aimed to describe the prevalence and incidence of drug-resistant tuberculosis and analyze its spatial, temporal, and spatiotemporal patterns in Paraná, Brazil, 2012–2023, and forecast trends through 2030. National surveillance data were analyzed using descriptive statistics, Mann–Kendall trend tests, Global and Local Moran’s I, Kulldorff’s spatial scan statistic, and Seasonal AutoRegressive Integrated Moving Average modeling. A total of 576 cases were identified, corresponding to an incidence of 5.08 per 100,000 inhabitants, with an increasing trend (*p* < 0.001). After peaking in 2019, incidence declined during the pandemic and rose in 2023. Isoniazid monoresistance was the most frequent profile. Prevalence was higher among males, young adults (15–34 years), and Asian and Black individuals. Spatial distribution showed expansion over time, with early circulation in the West. The North and Northwest exhibited an initial high incidence. Spatial and spatiotemporal analyses identified persistent high-risk clusters in these regions (*p* < 0.05). Forecasting suggests that, if current conditions persist, the incidence may continue to rise through 2030. The findings highlight the need for surveillance to ensure treatment adherence and interrupt transmission of resistant bacilli, supporting progress toward the global goal of tuberculosis elimination.

## 1. Introduction

The emergence and spread of drug-resistant tuberculosis (DR-TB) pose major challenges to achieving the targets set by the WHO’s End TB Strategy, which aims to eliminate the tuberculosis (TB) epidemic by 2030 through reducing cases, deaths, and catastrophic costs via actions tailored to diverse national contexts. Resistance to first-line drugs such as isoniazid (INH) and rifampicin (RIF), particularly in multidrug-resistant (MDR) cases, compromises treatment outcomes and contributes to sustained TB mortality, which has regained its position as the leading cause of death from a single infectious agent [1,2,3,4].

Despite not being among the 30 countries with the highest DR-TB burden, Brazil has a high TB burden and is considered at risk for the spread of resistant forms, which remain a major public health concern. Between 2015 and 2023, 17,200 new cases of DR-TB were reported in the country [4]. During 2023, 1060 cases were diagnosed, highlighting the importance of a decentralized strategy for laboratory diagnosis. These cases are subject to mandatory reporting and are monitored through the Unified Health System (SUS), which also ensures free access to treatment, follow-up, and public health surveillance, including contact tracing, active case finding, monitoring of treatment dropout, and appropriate case closure [5,6,7].

Confirmation of DR-TB cases requires drug susceptibility testing, which is carried out within a network of laboratories coordinated by the Brazilian Ministry of Health. In the subnational context of Paraná State, located in southern Brazil, the Central Public Health Laboratory (LACEN-PR) is responsible for performing drug susceptibility testing on all positive TB cultures from the region [8].

With advances in TB detection services, trend analysis and spatial-temporal approaches have become important tools to support the identification of DR-TB distribution patterns and enhance public health decision-making [9]. Paraná reported a TB incidence rate of 19.3 cases per 100,000 inhabitants in 2023, lower than the national incidence of 37.0 per 100,000 but still reflecting a substantial public health burden [7].

It is important to mention that investigations specifically focused on DR-TB patterns in Paraná remain scarce, limiting a broader understanding of how the disease behaves in this regional context. Expanding this knowledge is necessary to improve surveillance and guide more targeted interventions.

This study aimed to describe the prevalence and incidence of DR-TB and to analyze its spatial, temporal, and spatiotemporal patterns in Paraná, Brazil, from 2012 to 2023, as well as to forecast incidence trends through 2030.

## 2. Materials and Methods

### 2.1. Study Design and Scenario

This ecological study analyzed spatial, temporal, and spatiotemporal patterns of DR-TB from 2012 to 2023 in the state of Paraná, one of the 27 federal units in Brazil. Situated in the southern region, Paraná is bordered by the states of São Paulo, Mato Grosso do Sul, and Santa Catarina, and shares international borders with Paraguay and Argentina. Paraná has approximately 11.5 million inhabitants and is administratively organized into four health Macro-regions (East, Northwest, North, and West) and 22 Health Regions, which guide planning and management within the SUS across 399 municipalities (Table S1) [10]. The study followed STROBE guidelines [11].

### 2.2. Participants and Data Sources

Non-nominal records were collected in March 2025 from the Notifiable Diseases Information System (SINAN), available through the public platform of the Department of Informatics of SUS (https://datasus.saude.gov.br/, accessed on 19 March 2025), managed by the Brazilian Ministry of Health. Data are routinely entered by healthcare professionals using standardized notification forms, as defined in the national data dictionary (version 5.0) [12,13,14].

All TB cases reported as DR-TB among residents of Paraná were included. A case was defined as any TB notification with laboratory-confirmed resistance to one or more anti-TB drugs based on drug susceptibility testing (DST). Cases lacking DST results or unreported laboratory data were excluded and not used in the analyses (Figure S1).

The database provided four categories of resistance profiles: (i) resistance to isoniazid (INH^R^) only, classified as monoresistant; (ii) resistance to rifampicin (RIF^R^) only, also classified as monoresistant; (iii) multidrug-resistant TB (MDR-TB), defined as resistance to at least both INH and RIF; and (iv) Others, referring to resistance to other first-line drugs such as ethambutol and/or pyrazinamide, excluding isoniazid and rifampicin, but the specific drugs in this category were not detailed in the database (Figure S1).

The database was initially reviewed for the period from 1 January 2001, to 31 December 2023. However, as no DR-TB cases were recorded in the system before 2012, the years 2001 to 2011 were excluded due to absence of reported DR-TB cases. Thus, the final dataset comprised cases reported between 1 January 2012, and 31 December 2023. Population data from the 2022 Demographic Census, provided by the Paraná Institute of Economic and Social Development (IPARDES) [15] and based on Brazilian Institute of Geography and Statistics (IBGE) estimates, were used to calculate incidence rates (per 100,000 inhabitants) [16]. Cartographic shapefiles for the state of Paraná and its Health Regions were also obtained from IBGE [17]. The data sources are detailed in the Supplementary Material (Table S2).

Within Brazil’s TB diagnostic infrastructure, smear microscopy and culture are complemented by rapid molecular testing (RMT-TB) using the GeneXpert^®^ MTB/RIF Ultra system (Cepheid^®^, Sunnyvale, CA, USA), which detects *Mycobacterium tuberculosis* and genotypically identifies RIF^R^ with high sensitivity. The reverse hybridization line probe assay (LPA), using GenoType MTBDRplus (Hain Lifescience, Nehren, Germany), endorsed by the Ministry of Health, further identifies resistance-associated mutations, supporting timely therapeutic decisions. In Paraná, all TB-positive samples from the TB network are forwarded to LACEN-PR for confirmatory LPA testing under a centralized workflow, and before the incorporation of genotypic methods, phenotypic DST was routinely performed using Mycobacteria Growth Indicator Tube (MGIT) system (Becton Dickinson, Sparks, MD, USA), which remains available when necessary (Table S3) [18,19,20,21].

### 2.3. Data Analysis

#### 2.3.1. Descriptive and Prevalence

A descriptive analysis was conducted to characterize DR-TB cases by sex (female, male), age group (0–14, 15–34, 35–64, and ≥65 years), and ethnicity (White, Black, Asian, Mixed-race, Indigenous and not informed). Data were stratified by the state’s four Health Macro-regions.

Prevalence rates were calculated per 100,000 inhabitants. Prevalence Ratios (PR) with 95% confidence intervals (95% CI) were estimated using, for each variable, the category with the lowest prevalence as the reference. A PR > 1 indicates a higher prevalence of DR-TB relative to the reference, while a PR < 1 indicates a lower prevalence. CIs excluding 1.0 were considered statistically significant.

#### 2.3.2. Temporal Trend

Temporal trends in DR-TB incidence were evaluated using the Mann–Kendall test. A significance level of 5% was adopted, and the results were interpreted based on Kendall’s tau (τ) and *p*-values. Incidence rates were calculated per 100,000 inhabitants by quarter and health division (Health Macro-region and Health Region).

#### 2.3.3. Spatial

Crude incidence rates and rates smoothed via local empirical Bayesian estimation were analyzed to minimize instability in areas with small population or few reported cases [22]. The unit of analysis included all 399 municipalities of Paraná.

Spatial autocorrelation was assessed using Global Moran’s I, which measures the presence, strength, and direction of spatial clustering across the study area. The index ranges from −1 (dispersion) to +1 (clustering), with values near zero indicating randomness [23].

Local spatial autocorrelation was evaluated with Local Indicators of Spatial Association (LISA), identifying statistically significant clusters among neighboring municipalities. Clusters were classified into four categories: High-High (hotspots), where municipalities with high DR-TB incidence are surrounded by others with similarly high rates; Low-Low (coldspots), where low-incidence areas are surrounded by low rates; High-Low, indicating high-incidence areas neighboring low ones (potential spatial outliers or transition zones); and Low-High, low-incidence municipalities adjacent to high-risk areas, also suggestive of transitional contexts. Spatial weights were defined using a first-order Queen contiguity matrix, and statistical significance was set at 5%, based on 999 Monte Carlo permutations [24].

#### 2.3.4. Spatiotemporal

To assess whether the spatial clustering of DR-TB cases was due to random variation over time, a retrospective space–time scan was performed using the Kulldorff method with the discrete Poisson probability model and quarterly aggregation (3-month intervals). The cylindrical scanning window moved across both space and time, with the base representing geographic units (Health Region) and the height representing time [25].

The maximum spatial cluster size was set to 50% of the population at risk, and the temporal window varied throughout the study period (2012–2023). Statistical significance was assessed using 999 Monte Carlo replications with a *p*-value threshold of 0.05. Clusters were classified based on their relative risk (RR) and log-likelihood ratio (LLR), comparing observed versus expected case counts under the null hypothesis of random distribution. Overlapping clusters were not allowed [26].

#### 2.3.5. Forecasting

The quarterly time series of DR-TB incidence was modeled using a Seasonal AutoRegressive Integrated Moving Average (SARIMA) model, expressed as SARIMA(*p*, *d*, *q*)(*P*, *D*, *Q*)[*m*], to forecast incidence trends through the year 2030 with 95% CI. In this specification, (*p*, *d*, *q*) represent the non-seasonal component (autoregressive order, differencing, and moving average order, respectively), while (*P*, *D*, *Q*)[*m*] define the seasonal component (seasonal autoregressive order, seasonal differencing, seasonal moving average order, and the seasonal cycle length in number of observations). This approach enables modeling both short-term fluctuations and recurring seasonal patterns, which are essential for understanding epidemiological time series. SARIMA models have been widely applied to forecast TB and other infectious disease trends in previous studies [27,28,29,30].

Prior to modeling, the series was smoothed using a Kalman filter to reduce volatility and observational noise, estimating the underlying signal and preventing the model from fitting spurious fluctuations. The dataset was partitioned into training and testing subsets: the training set covered March 2012 to June 2021, while the testing set included September 2021 to February 2023. Constraints enforcing stationarity and invertibility were applied to ensure stable and uniquely defined parameter estimates, while model parameters were optimized using the Powell algorithm with a maximum of 200 iterations. An exhaustive grid search was conducted over 1728 candidate parameterizations to identify the optimal SARIMA specification. The search tested non-seasonal parameters *p*, *d*, *q* ∈ {0,1,2,3} and seasonal parameters *P*, *D*, *Q* ∈ {0,1,2}, with the seasonal cycle length fixed at four to reflect quarterly periodicity. Model performance was evaluated using the Mean Absolute Percentage Error (MAPE), and predictive accuracy in the test set was prioritized over in-sample fit for parameters selection.

### 2.4. Software

Analyses were performed using RStudio (v4.4.3, Posit Software, Boston, MA, USA) for time-series, Python (v3.10, Python Software Foundation, Beaverton, OR, USA) for forecasting, GeoDa (v1.22.0.4, University of Chicago, Chicago, IL, USA) for spatial autocorrelation, and SaTScan (v10.3.2, Martin Kulldorff, Information Management Services, Inc., Rockville, MD, USA) for spatiotemporal analysis. Thematic maps were developed in QGIS (v3.36.0-0, QGIS Development Team, 2024).

### 2.5. Ethical Statement

This study was approved by the Research Ethics Committee of the State University of Maringá, Paraná, Brazil (approval number 6964500), in accordance with Resolution No. 466/2012 of the Brazilian National Health Council.

## 3. Results

### 3.1. Descriptive and Prevalence Analysis

**Figure 1:**
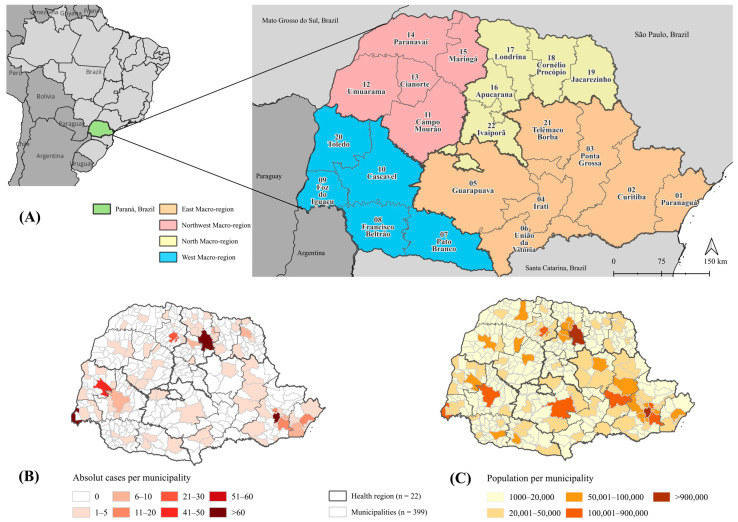
Spatial distribution of drug-resistant tuberculosis cases and population, Paraná, Brazil, 2012–2023. Note. (**A**) Territorial division of Paraná, Brazil, Health Macro-regions (*n* = 4) and Health Regions (*n* = 22). (**B**) Absolute drug-resistant tuberculosis cases per municipality (2012–2023). Irati’s Health Region 04 did not report any cases during the study period. (**C**) Absolute population per municipality based on census data.

Between 2012 and 2023, a total of 576 DR-TB cases were reported in Paraná. The highest number occurred in 2019 (*n* = 93; 16.1%) and 2023 (*n* = 69; 12.0%). Across Health Macro-regions the North (*n* = 177; 30.7%) and East (*n* = 176; 30.6%) showed the highest concentrations. At the sub-regional level, Londrina’s Health Region 17 recorded the most cases (*n* = 153; 26.6%), followed by Curitiba’s Health Region 02 (*n* = 123; 21.4%), Foz do Iguaçu’s Health Region 09 (*n* = 68; 11.8%), Toledo’s Health Region 20 (*n* = 58; 10.1%), and Maringá’s Health Region 15 (*n* = 57; 9.9%). Together, these five regions accounted for 79.7% of all cases during the study period (Figure 1).

**Figure 2:**
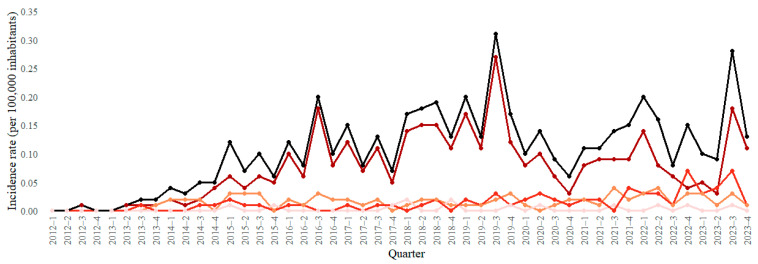
Trends in drug-resistant tuberculosis incidence rates by resistance profile, Paraná, Brazil, 2012–2023. Note. DR-TB: drug-resistant tuberculosis; INH^R^: isoniazid-resistant; RIF^R^: rifampicin-resistant; MDR-TB: multidrug-resistant tuberculosis, defined as resistance to at least isoniazid and rifampicin; Others: resistance to other first-line drugs (e.g., ethambutol and pyrazinamide).

Regarding sociodemographic characteristics (Table 1), most cases occurred in men (*n* = 444; 77.1%). White individuals predominated (*n* = 341; 59.2%), followed by Mixed-race (*n* = 163; 28.3%) and Black (*n* = 47; 8.2%) individuals. Cases were mainly concentrated in the 15–34 (*n* = 288; 50.0%) and 35–64 (*n* = 252; 43.8%) age groups. In terms of resistance profiles, 72.0% were INH^R^, 12.5% RIF^R^, 13.2% MDR-TB, and 2.3% resistant to other first-line drugs (Figure 2).

By individual characteristics, a consistent pattern was observed across all Health Macro-regions, with higher prevalence among males (PR = 3.538; 95% CI: 3.224–3.883) and young adults aged 15–34 years (PR = 23.444; 95% CI: 11.613–47.330). At the state level, Asian (PR = 2.594; 95% CI: 1.459–4.613) and Black individuals (PR = 2.097; 95% CI: 1.546–2.844) showed higher prevalence compared to White individuals, whereas the increases among Indigenous and mixed-race groups did not reach statistical significance (Table 1).

### 3.2. Temporal Trend Analysis

In terms of caseload, the North (177; 30.73%) and East (176; 30.56%) Macro-regions accounted for the largest shares during the period. The highest cumulative incidence was observed in Foz do Iguaçu’s Health Region 9, at 16.85 cases per 100,000 inhabitants. Paraná exhibited a statistically significant increasing trend (*p* < 0.001). Trends were also rising across all Macro-regions, with the most pronounced increases in the North and Northwest (both *p* < 0.001). Among Health Regions, significant increases were found in Londrina’s Health Region 17 (*p* < 0.001), Maringá’s Health Region 15 (*p* < 0.001), Pato Branco’s Health Region 07 (*p* = 0.014), Umuarama’s Health Region 12 (*p* = 0.035), and Curitiba’s Health Region 02 (*p* = 0.029), three of which are located in the Northern and Northwestern Macro-regions (Table 2).

### 3.3. Spatial Analysis

**Figure 3:**
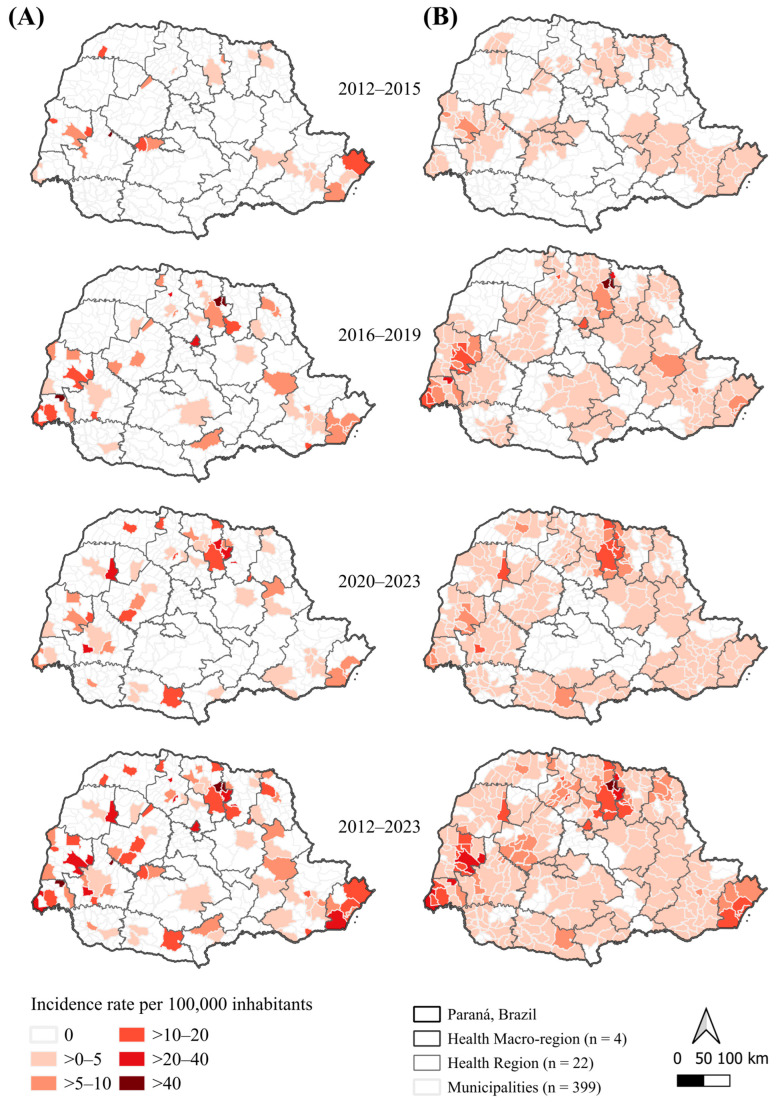
Spatial distribution of crude and smoothed drug-resistant tuberculosis incidence rates by municipality and four-year periods, Paraná, Brazil, 2012–2023. Note. (**A**) Crude incidence rates (per 100,000 inhabitants). (**B**) Empirical Bayes–smoothed incidence rates (per 100,000 inhabitants).

Territorial expansion of DR-TB incidence was observed across four-year periods (Figure 3). During 2012–2015, only a few municipalities, mainly in the West and North, presented moderate incidence rates (10–20 cases per 100,000 inhabitants). From 2016–2019 onward, incidence became increasingly concentrated in the North, Northwest, and West Macro-regions. By 2020–2023, several municipalities exceeded 20 cases per 100,000, with some surpassing 40 per 100,000, particularly in the Northwest. Smoothed maps (Figure 3B) highlighted more stable spatial patterns and persistent hotspots in Health Regions of Londrina, Maringá, and Toledo, while low-incidence areas remained concentrated in the East.

**Figure 4:**
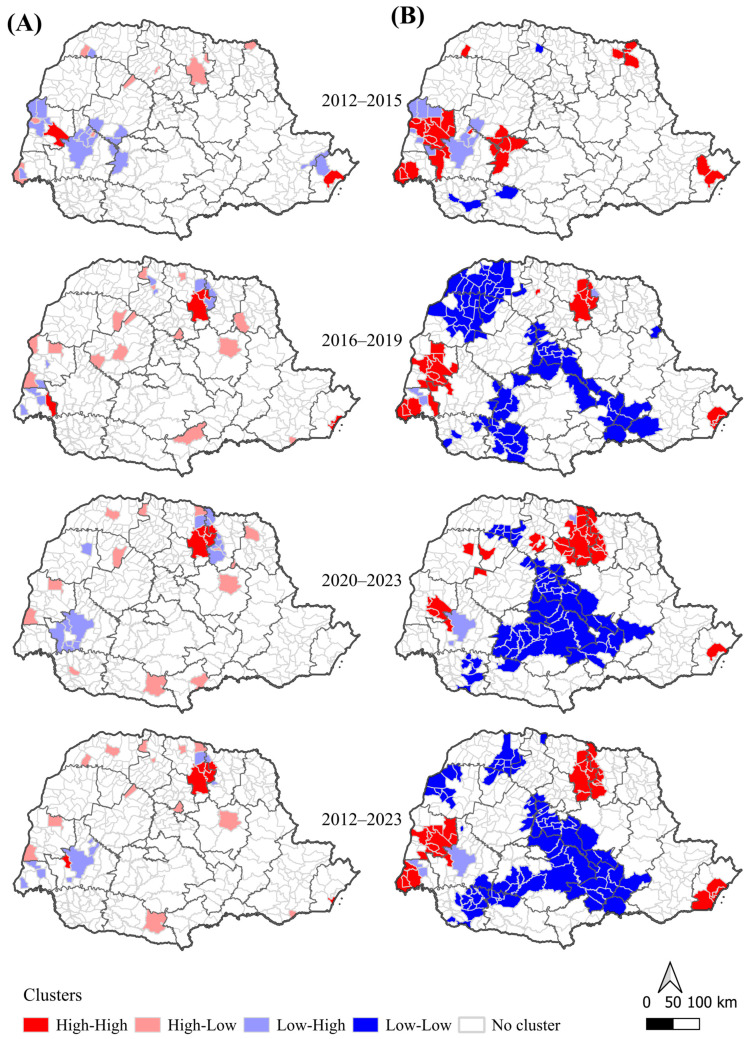
Local spatial autocorrelation (LISA) for crude and smoothed drug-resistant tuberculosis incidence rates by municipality and four-year periods, Paraná, Brazil, 2012–2023. Note. (**A**) Crude incidence rates (per 100,000 inhabitants). (**B**) Empirical Bayes-smoothed incidence rates (per 100,000 inhabitants). Clusters (*p* < 0.05): High–High denote areas with high incidence surrounded by high-incidence neighbors (hotspots); Low–Low denote low incidence surrounded by low-incidence neighbors (coldspots); High–Low and Low–High denote transitional areas between high- and low-incidence regions.

Global spatial dependence was statistically significant for smoothed rates (*p* < 0.01), with Moran’s I increasing from 0.272 (2012–2015) to 0.522 (2020–2023), and reaching 0.510 for the overall period (2012–2023). Although moderate, these values indicate a consistent intensification of spatial clustering. LISA results (Figure 4) confirmed a progressive consolidation and expansion of High–High clusters in the North and Northwest, and the stability of Low–Low clusters in the East.

### 3.4. Spatiotemporal Analysis

**Figure 5:**
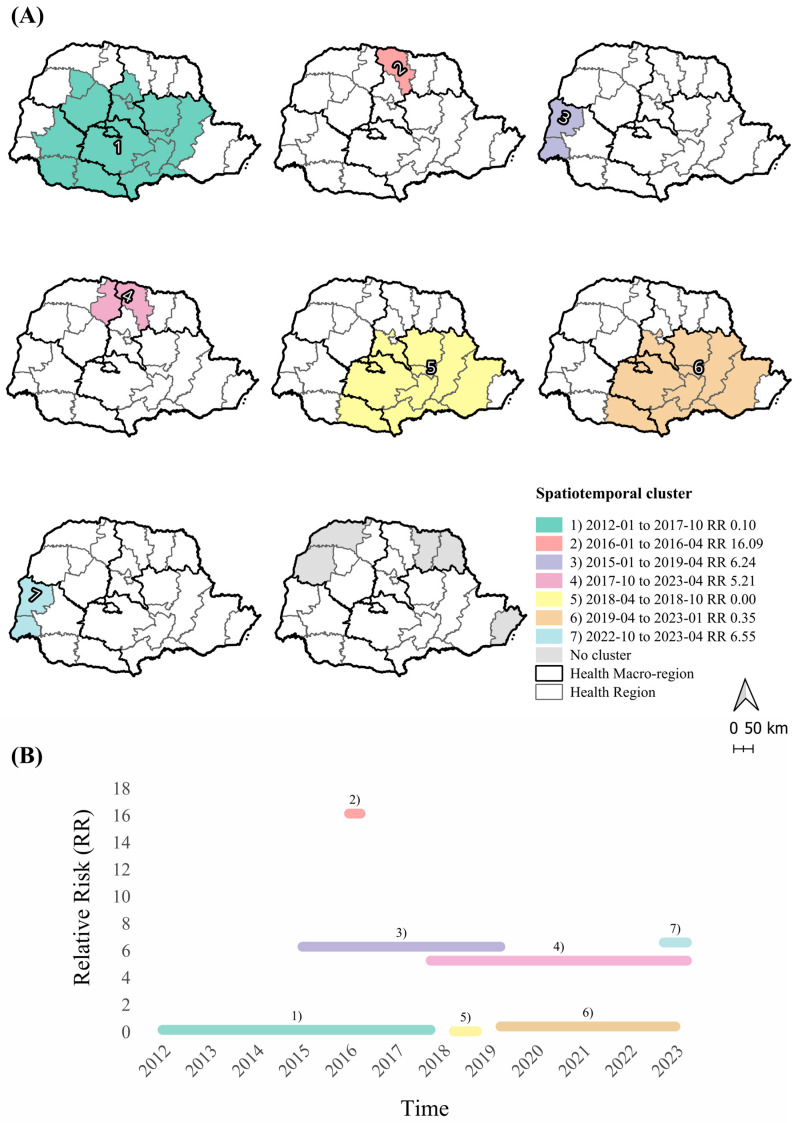
Space–time clusters of drug-resistant tuberculosis by Health Region, Paraná, Brazil, 2012–2023. Note. (**A**) Spatiotemporal clusters of drug-resistant tuberculosis in Paraná, Brazil (2012–2023), with relative risk (RR) and period of occurrence (*p* < 0.05). Cluster 1: Health Regions 03; 04; 05; 06; 07; 08; 10; 11; 13; 16; 21; 22. Cluster 2: Health Region 17. Cluster 3: Health Regions 09; 20. Cluster 4: Health Regions 15; 17. Cluster 5: Health Regions 02; 03; 04; 05; 06; 07; 21; 22. Cluster 6: Health Regions 02; 03; 04; 05; 06; 07; 21; 22. Cluster 7: Health Regions 09; 20. (**B**) Duration and RR of clusters over time.

Space–time cluster analysis identified two main statistically significant clusters (*p* < 0.05) (Figure 5, Table 3). The primary high-risk cluster included Maringá’s Health Region 15 and Londrina’s Health Region 17, from October 2017 to April 2023, where the risk of DR-TB was more than five times higher compared to other regions (RR 5.21; *p* = 0.001). The secondary cluster involved Foz do Iguaçu’s Health Region 9 and Toledo’s Health Region 20, between January 2015 and April 2019, with a more than sixfold increased risk (RR 6.24; *p* = 0.001).

A short-duration cluster was detected in Londrina’s Health Region 17 from January to April 2016, presenting the highest observed risk, over 16 times greater than in other areas (RR 16.09; *p* = 0.001). Additional clusters with risks exceeding sixfold were detected between October 2022 and April 2023. A low-risk cluster (*p* = 0.017) was found in Eastern Health Regions. These findings corroborate the increasing trends observed in Londrina’s Health Region 17 and Maringá’s Health Region 15, demonstrating a convergence between spatial and temporal risk patterns.

### 3.5. Forecast

The SARIMA(0,2,3)(1,0,1) [4] model was identified as the optimal model following an exhaustive grid search over 1728 candidate parameterizations. Model selection was based on predictive accuracy, with the lowest MAPE (11.5%) observed in the test dataset (Figure S2).

**Figure 6:**
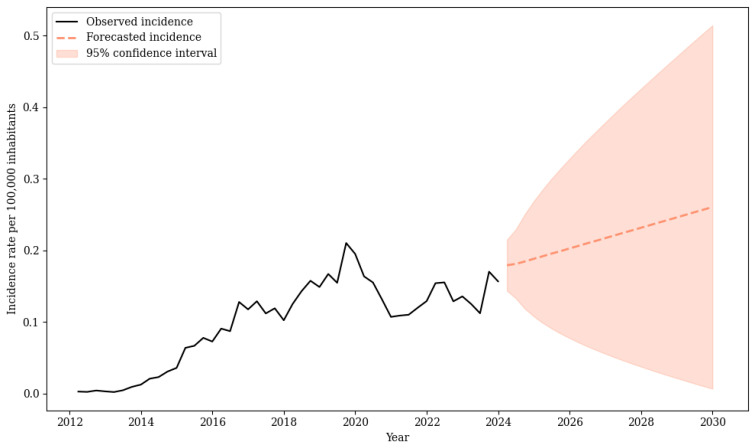
Forecast of drug-resistant tuberculosis incidence rate, Paraná, Brazil, 2024–2030.

The selected model was then applied to forecast the next 28 quarters (2024–2030), providing point estimates along with 95% CI to capture both the projected trend and the associated uncertainty. Results indicate an increasing trend, from 0.179 (95% CI: 0.143–0.215) in 2024 to 0.260 (95% CI: 0.006–0.514) in 2030, corresponding to an estimated 620 DR-TB cases (95% CI: 169–1071), if the current scenario remains unchanged (Figure 6).

## 4. Discussion

This study provided a detailed overview of the spatial high-risk regions and temporal trends of DR-TB, revealing persistent and intensifying clusters over time, particularly in the Northern and Northwestern Health Macro-regions of Paraná. The overall DR-TB rate increased until 2019, followed by a reduction during 2020 and 2021, coinciding with the impact of the COVID-19 pandemic on global TB control efforts, affecting diagnosis and case reporting [4]. From 2022 onwards, rates fluctuated, with renewed growth observed in 2023. Projections indicate that, if no additional interventions are implemented, DR-TB incidence may continue to rise through 2030.

DR-TB may result from primary infection with resistant bacilli or, more commonly, from acquired resistance driven by inadequate treatment, inappropriate drug regimens, or therapy interruption. These factors promote the selection and dissemination of resistant *M. tuberculosis*, especially in settings with high rates of treatment default. Inadequate management of these cases not only facilitates the emergence of new resistant mutations but also increases the risk of ongoing transmission and further infections with resistant strains [4,31,32].

The implementation of rapid molecular testing in 2014, followed by the progressive expansion of the TB diagnostic network in Paraná, likely improved the detection and reporting of DR-TB cases across the state. This pattern was also observed at the national level, where a previous study reported an 80% increase in the detection of resistance in municipalities equipped with this technology [33]. The recent introduction of the LPA in 2021 further strengthened laboratory confirmation and reduced diagnostic delays. Therefore, the observed increase in notifications after 2014 may reflect enhanced detection capabilities rather than a true rise in incidence, as also reported in other Brazilian studies [8,34,35,36,37]. Nevertheless, a genuine rise in incidence cannot be ruled out, as the persistence of upward trends beyond the initial diagnostic scale-up suggests that both epidemiological changes and strengthened surveillance may be contributing simultaneously.

The temporal analysis revealed a significant increase in DR-TB in the Health Regions of Londrina (North), Maringá and Umuarama (Northwest), Pato Branco (West), and Curitiba (East), reflecting a concentrated growth pattern in these areas. In contrast, Foz do Iguaçu and Toledo (West) maintained high incidence rates, but did not exhibit statistically significant trends. At the state level, an overall upward trajectory of DR-TB was observed, indicating that sustained and regionally concentrated increases call for targeted surveillance and resource allocation to address the growing burden of DR-TB and highlighting the intensification of the problem over the last decade.

Regarding the resistance profile during the study period, INH monoresistance was the most frequent pattern among *M. tuberculosis isolates* circulating in Paraná, followed by RIF. This result contrasts with the national scenario, where RIF^R^ and MDR-TB predominate in most Brazilian states [35,38,39]. Internationally, INH^R^ is also predominant in several middle-income countries, such as India, and Tanzania [2,40], while in settings like South Africa, Russia, and Eastern Europe, RIF^R^ and MDR-TB prevail [1,41]. This distribution may be linked to historical treatment practices and local transmission dynamics but aligns with broader trends described in other countries.

Spatial analysis revealed territorial expansion and the consolidation of persistent hotspots in the North, Northwest, and West Macro-regions, while the East remained a low-risk area. These high-risk clusters may reflect increased DR-TB transmission, greater circulation of resistant strains, or enhanced surveillance sensitivity, which facilitates the identification of resistant cases. Moreover, treatment non-adhesion is a critical factor that can favor the emergence and amplification of drug-resistant TB, particularly in high-burden regions [40,41,42,43,44].

Spatiotemporal analysis further confirmed these spatial patterns. The first low-risk cluster, covering parts of the East and West Macro-regions, reflected an initial phase of DR-TB detection with fewer cases than expected or may indicate only an early stage of laboratory surveillance rather than an actual absence of resistant cases [19]. These low-incidence municipalities, located near high-risk areas, may reflect actual lower transmission or, alternatively, point to potential underreporting and reduced diagnostic reach.

Notably, significant high-risk clusters in the North and Northwest Macro-regions are situated near the borders with other Brazilian states, particularly São Paulo, which also reports a high burden of TB and DR-TB cases [39,45], as well as in international border regions such as Foz do Iguaçu, located in the tri-border area of the West Macro-region. The eastern coastal area, including Paranaguá’s Health Region 01, was identified as a high-high cluster at different time periods. Given its role as a major maritime port, combined with social vulnerabilities and persistently high TB incidence [45], this region requires strengthened DR-TB surveillance.

From an epidemiological perspective, these areas pose unique challenges due to intense cross-border interactions, as migration flows, precarious living conditions, and differences in healthcare access between countries contribute to increased TB and DR-TB burden in frontier regions. Evidence suggests that both local transmission and international migration contribute to TB occurrence in these border areas, and that outbreaks in the community and prison systems are often interrelated. Studies have demonstrated the circulation of similar *M. tuberculosis* genotypes, including multidrug-resistant strains, in Brazil’s international border regions, including those with Paraguay and Argentina. In this scenario, TB may spread across Brazilian borders as constant migratory movements, combined with adverse social conditions, may facilitate both the maintenance of local transmission chains and the introduction of new genotypes from neighboring countries [31,32,46,47,48,49]. Since the transmission of bacilli is not constrained by territorial boundaries, strengthening surveillance and coordinated actions is essential for controlling and preventing new cases.

Men exhibited a prevalence more than three times higher than that of women, and young adults were the most affected age group, consistent with national and international findings. This disparity may be partially explained by the greater exposure of these groups to social vulnerabilities such as homelessness, incarceration, regular alcohol and illicit drug use, and HIV infection. These factors are strongly associated with treatment interruption/loss to follow-up, which in turn increases the risk of acquired drug resistance and facilitates the transmission of resistant strains [2,50,51,52,53,54].

Although national data indicate a higher absolute burden of DR-TB among Black populations [52], in Paraná the majority of cases occurred among Whites, reflecting the state’s demographic profile marked by a predominance of European-descendant groups. However, PR was elevated among Black and Asian populations, which may be partly related to the fact that Asians represent a larger share of the state’s population compared with the national average [16].

In addition to microbiological aspects, DR-TB imposes a substantially higher economic burden than drug-susceptible TB, owing to longer treatment duration and higher indirect costs, such as work absenteeism and income loss. Globally, households affected by DR-TB are more likely to experience catastrophic costs [4]. In Brazil and other middle-income countries with a high TB burden, participation in conditional cash transfer programs has been associated with improved treatment success, reduced dropout, and lower mortality rates [55,56].

Social and structural determinants, such as poverty, incarceration, and limited access to health services, have been associated with the burden of DR-TB. These interrelated factors may help explain the persistence and intensification of high-risk clusters in the North, Northwest, and West Macro-regions, despite the expansion of diagnostic coverage. A study in a prison population in Paraná showed a growing trend of cases, highlighting that correctional facilities have become environments conducive to the persistence of both TB and resistant forms of the disease [32]. In particular, previous inadequate treatment, HIV coinfection, and substance abuse are well-established and interrelated risk factors that facilitate the development and transmission of resistant strains, especially when combined with other comorbidities and vulnerabilities, including barriers to accessing health services [40,41,42,43,44]. These interdependent social and biomedical determinants interact to sustain the transmission of bacilli and complicate efforts to ensure treatment adherence, and increase the risk of unfavorable outcomes.

A Brazilian survey on healthcare coverage revealed that less than 30% of the population have private insurance, reinforcing the role of SUS as the main healthcare provider. Primary care and basic health units remain essential pillars of public health for TB and DR-TB diagnosis and monitoring of treatment [57]. Despite national guidelines recommending standardized treatment regimens, directly observed therapy, and the inclusion of social protection strategies, disparities in access persist, particularly among socially and economically vulnerable groups, who often face unmet needs for essential health services and medications [58].

Marked regional and socioeconomic disparities remain, even in states with high coverage rates such as Paraná [59]. Historically, the phenomenon of inverse equity has been observed in the state, with primary healthcare services concentrated in municipalities with more favorable socioeconomic and structural conditions. Differences in access between Macro-regions could present challenges to the uniform implementation of TB and DR-TB control strategies, and may also contribute to the spread of other diseases [60,61].

The forecast indicates that, without targeted and sustained efforts to interrupt DR-TB, the incidence burden in the state may worsen in the coming years. These results should be interpreted as conditional scenarios, since they assume the continuation of past trends. The projections are accompanied by 95% confidence intervals, which widen over time, indicating that the estimates should be interpreted as indicative trends rather than precise predictions [27,30]. Potential programmatic innovations (e.g., introduction of new treatment regimens, expanded molecular testing, or strengthened case-finding strategies) may modify future trajectories. However, this underscores the importance of an active public health surveillance system capable of early detection of resistant cases, monitoring high-risk clusters, and guiding timely interventions to achieve the End TB strategy targets aligned with the United Nations Sustainable Development Goal 3 [4,62].

Despite advances in rapid molecular diagnostics and shorter treatment regimens, expanding access to diagnostic technologies and strengthening health infrastructure remain essential, particularly in high-risk areas and persistent clusters identified in this regional study. Furthermore, targeted surveillance and laboratory diagnostics are critical for preventing the introduction and dissemination of highly resistant strains, which is a global concern, especially given the limited availability of effective therapeutic options and poorer clinical outcomes [4,19].

This study has some limitations that should be acknowledged. Although underreporting, misclassification, or missing variables may occur with secondary data [63], this system remains essential for TB surveillance in Brazil. The long time series enabled the identification of relevant DR-TB patterns, despite the ecological design and aggregated units (Macro- and Health Regions), which restrict causal inference and may conceal local heterogeneity in incidence and transmission dynamics. Regional-level analysis was chosen to reduce statistical instability and enhance decision-making relevance within Paraná’s health system. Wider confidence intervals in the prevalence analysis may reflect reduced sample sizes in some subgroups. The database used in this study does not distinguish between acquired and primary drug resistance and provides limited information on drugs other than INH and RIF, and therefore does not contain sufficient detail to classify cases as pre-XDR (MDR-TB with additional resistance to any fluoroquinolone) or XDR-TB (pre-XDR plus resistance to at least one Group A drug, e.g., bedaquiline or linezolid). Future research should integrate contextual factors to clarify spatial patterns and potential drivers of DR-TB distribution, as well as investigate the resistance patterns of clinical isolates in greater detail.

## 5. Conclusions

Current trends and projections suggest the persistence of DR-TB in Paraná, which may lead to higher healthcare costs, increased clinical complexity, and potentially greater mortality. Addressing this requires sustained vigilance, timely diagnosis, treatment adhesion, and focused monitoring in high-risk areas identified through spatial analysis. Geographically targeted strategies are essential to contain resistant bacilli and reduce future disease burden. The findings can guide surveillance and resource allocation, emphasizing targeted actions in high-risk and neighboring areas, including enhanced cross-border surveillance and prioritization of resources to hotspots, to improve outcomes and prevent the spread of resistant *M. tuberculosis*, thereby supporting progress toward the End TB goals.

## Figures and Tables

**Table 1:** Prevalence of drug-resistant tuberculosis by sex, ethnicity, and age group across Health Macro-regions and the state of Paraná, Brazil (2012–2023).

	Health Macro-Region																State of Paraná			
	East				North				Northwest				West							
Variables	*n*	Pop.	Prev.	PR (95% CI)	*n*	Pop.	Prev.	PR (95% CI)	*n*	Pop.	Prev.	PR (95% CI)	*n*	Pop.	Prev.	PR (95% CI)	*n*	Pop.	Prev.	PR (95% CI)
Sex																				
Female	58	2,799,061	2.072	Ref	30	1,009,428	2.972	Ref	9	987,243	0.912	Ref	35	1,071,298	3.267	Ref	132	5,867,030	2.250	Ref
Male	118	2,658,318	4.439	2.142 (1.564–2.933) *	147	952,442	15.434	5.193 (3.507–7.691) *	67	936,408	7.155	7.849 (3.914–15.739) *	112	1,030,182	10.872	3.328 (2.277–4.864) *	444	5,577,350	7.961	3.538 (3.224–3.883) *
Ethnicity																				
White	123	3,721,165	3.305	Ref	106	1,225,505	8.649	Ref	31	1,094,920	2.831	Ref	81	1,348,342	6.007	Ref	341	7,389,932	4.614	Ref
Black	7	212,933	3.287	0.995 (0.464–2.130)	18	102,282	17.598	2.035 (1.235–3.353) *	13	97,420	13.344	4.713 (2.466–9.007) *	9	73,146	12.304	2.048 (1.029–4.078) *	47	485,781	9.675	2.097 (1.546–2.844) *
Asian	6	33,050	18.154	5.492 (2.420–12.463) *	4	33,175	12.057	1.394 (0.514–3.783)	1	26,470	3.778	1.334 (0.182–9.774)	1	7549	13.247	2.205 (0.307–15.843)	12	100,244	11.971	2.594 (1.459–4.613) *
Mixed-race	36	1,479,987	2.432	0.736 (0.508–1.067)	46	593,168	7.755	0.897 (0.634–1.267)	29	703,486	4.122	1.456 (0.878–2.416)	52	663,396	7.838	1.305 (0.921–1.848) *	163	3,440,037	4.738	1.027 (0.852–1.238)
Indigenous	-	10,025	-	-	0	7696	-	-	0	1291	-	-	2	8988	22.252	3.704 (0.911–15.063)	2	28,000	7.143	1.548 (0.386–6.214)
Not informed	4	219	-	-	3	44	-	-	2	64	-	-	2	59	-	-	11	386	-	-
Age group																				
0–14	5	1,059,932	0.472	Ref.	2	360,890	0.554	Ref.	1	357,909	0.279	Ref.	0	416,566	-	-	8	2,195,297	0.364	Ref.
15–34	76	1,645,667	4.618	9.790 (3.961–24.198) *	108	549,396	19.658	35.472 (8.759–143.658) *	44	544,005	8.088	28.948 (3.988–210.115) *	60	631,948	9.494	2.716 (1.299–5.679) *	288	3,371,016	8.543	23.444 (11.613–47.330) *
35–64	88	2,183,659	4.030	8.543 (3.469–21.035) *	58	791,791	7.325	13.218 (3.228–54.119) *	27	779,621	3.463	12.395 (1.684–91.220) *	79	824,126	9.586	2.742 (1.325–5.674) *	252	4,579,197	5.503	15.101 (7.470–30.528) *
≥65	7	568,121	1.232	2.612 (0.829–8.230)	9	259,793	3.464	6.251 (1.651–28.932) *	4	242,116	1.652	5.913 (0.661–52.905)	8	228,840	3.496	Ref	28	1,298,870	2.156	5.916 (2.696–12.979) *

Note. Absolute cases (*n*); exposed population (Pop.); prevalence per 100,000 inhabitants (Prev.); Prevalence Ratios (PR) calculated using the lowest-prevalence category as reference (Ref.); 95% CI not including 1.0 indicates a statistically significant association (*); “Not informed” denotes missing data.

**Table 2:** Temporal trends in drug-resistant tuberculosis incidence rate by Health Macro-region and Health Region, Paraná, Brazil, 2012–2023.

Health Division	*n*	(%)	Incidence Rates	95% CI	Kendall τ	*p*-Value	Trends
East Macro-region	176	30.56	3.18	2.71–3.65	0.2896	0.006 *	Increasing
01 Paranaguá	35	6.08	11.90	7.96–15.84	0.1431	0.197	Stationary
02 Curitiba	123	21.35	3.40	2.80–4.00	0.2295	0.029 *	Increasing
03 Ponta Grossa	9	1.56	1.42	0.49–2.35	0.2001	0.094	Stationary
05 Guarapuava	4	0.69	0.88	0.02–1.74	−0.0772	0.536	Stationary
06 União da Vitória	2	0.35	1.13	−0.44–2.70	0.1304	0.290	Stationary
21 Telêmaco Borba	3	0.52	1.60	−0.21–3.41	0.1145	0.349	Stationary
West Macro-region	147	25.52	7.47	6.26–8.68	0.2541	0.015 *	Increasing
07 Pato Branco	4	0.69	1.51	0.03–2.97	0.2940	0.014 *	Increasing
08 Francisco Beltrão	2	0.35	0.56	−0.22–1.34	0.1019	0.409	Stationary
09 Foz do Iguaçu	68	11.81	16.85	12.84–20.86	0.1193	0.266	Stationary
10 Cascavel	15	2.60	2.74	1.35–4.13	0.1365	0.244	Stationary
20 Toledo	58	10.07	14.69	10.91–18.47	0.0436	0.688	Stationary
Northwest Macro-region	76	13.19	4.07	3.16–4.98	0.5546	<0.001 *	Increasing
11 Campo Mourão	9	1.56	2.73	0.95–4.51	0.1332	0.263	Stationary
12 Umuarama	6	1.04	2.18	0.44–3.92	0.2535	0.035 *	Increasing
13 Cianorte	2	0.35	1.26	−0.48–3.00	0.0931	0.454	Stationary
14 Paranavaí	2	0.35	0.73	−0.28–1.74	−0.0340	0.797	Stationary
15 Maringá	57	9.90	6.88	5.09–8.67	0.4851	<0.001 *	Increasing
North Macro-region	177	30.73	8.94	7.62–10.26	0.6190	<0.001 *	Increasing
16 Apucarana	6	1.04	1.58	0.32–2.84	0.1950	0.106	Stationary
17 Londrina	153	26.56	16.00	13.46–18.54	0.5767	<0.001 *	Increasing
18 Cornélio Procópio	6	1.04	2.69	0.54–4.84	0.1370	0.252	Stationary
19 Jacarezinho	11	1.91	3.81	1.56–6.06	0.0780	0.513	Stationary
22 Ivaiporã	1	0.17	0.77	−0.74–2.28	0.0651	0.613	Stationary
Paraná	576	100.00	5.08	4.67–5.49	0.5183	<0.001 *	Increasing

Note. Absolute number of cases (*n*); frequency (%); incidence rate per 100,000 inhabitants (Incidence); 95% confidence interval (95% CI); Mann–Kendall test (τ and *p*-value). Asterisks (*) denote statistically significant increasing trends (*p* < 0.05). Population estimates are based on census data. Irati’s Health Region 04 reported no cases during the study period.

**Table 3:** Spatiotemporal clusters of drug-resistant tuberculosis by Health Region, Paraná, Brazil, 2012–2023.

Cluster	Health Region Included	Time Frame	Observed Cases	Expected Cases	Crude Incidence Rate	LLR	RR	Cluster Population	*p*-Value
1	03; 04; 05; 06; 07; 08; 10; 11; 13; 16; 21; 22	2012-01–2017-10	11	93.83	0.05	65.99	0.10	3,796,109	0.001
2	17	2016-01–2016-04	16	1.02	6.8	29.25	16.09	946,055	0.001
3	09; 20	2015-01–2019-04	81	14.71	2.4	75.96	6.24	807,908	0.001
4	15; 17	2017-10–2023-04	171	43.21	1.7	124.16	5.21	1,770,577	0.001
5	02; 03; 04; 05; 06; 07; 21; 22	2018-04–2018-10	0	12.31	0.0	12.43	0.00	5,577,542	0.017
6	02; 03; 04; 05; 06; 07; 21; 22	2019-04–2023-01	36	92.50	0.2	25.70	0.35	5,577,542	0.001
7	09; 20	2022-10–2023-04	12	1.86	2.8	12.30	6.55	3,796,109	0.019

Note. Clusters (*p* < 0.05); Period: start–end (year/quarter); Relative risk (RR); Log-likelihood ratio (LLR); Crude incidence rate (per 100,000 inhabitants); Cluster population refers to the total estimated population in the involved Health Regions during the corresponding time frame.

## Data Availability

All the data used can be downloaded, aggregated by municipality, from: Ministry of Health of Brazil, Notifiable Diseases Information System (SINAN). Available online: https://datasus.saude.gov.br/transferencia-de-arquivos/ (accessed on 19 March 2025). The datasets are also available from the corresponding author upon request via email.

## Supplementary Materials

The following supporting information can be downloaded at: https://www.mdpi.com/article/10.3390/pathogens14101046/s1. Figure S1. Flowchart of case selection for drug-resistant tuberculosis, Paraná, Brazil, 2012–2023; Figure S2. Seasonal–trend decomposition of the quarterly incidence rate of drug-resistant tuberculosis, Paraná, Brazil, 2012–2023; Table S1. Political-Administrative Division of Paraná State, Brazil; Table S2. Data sources, definitions, and links for indicators included in the analysis; Table S3. Diagnostic algorithm for tuberculosis according to the Manual of the Brazilian Ministry of Health.

## Abbreviations

The following abbreviations are used in this manuscript:

DATASUS	Department of Informatics of the Brazilian Unified Health System
DR-TB	Drug-resistant tuberculosis
IBGE	Brazilian Institute of Geography and Statistics
IPARDES	Paraná Institute for Economic and Social Development
INH	Isoniazid
INH^R^	Isoniazid resistance
LACEN-PR	Central Public Health Laboratory of Paraná
LISA	Local Indicators of Spatial Association
LPA	Line Probe Assay
MDR-TB	Multidrug-resistant tuberculosis
PR	Prevalence Ratio
pre-XDR-TB	Pre-extensively drug-resistant tuberculosis
RR	Relative risk
SARIMA	Seasonal Autoregressive Integrated Moving Average
SINAN	Notifiable Diseases Information System
SUS	Universal Public Healthcare System/Unified Health System
TB	Tuberculosis
RIF	Rifampicin
RIF^R^	Rifampicin resistance
XDR-TB	Extensively drug-resistant tuberculosis
WHO	World Health Organization
