# Supplementary material for: From Patterns to Projections: A Spatiotemporal Distribution of Drug-Resistant Tuberculosis in Paraná, Brazil (2012–2023)
Source: Pathogens. 2025 Oct 16;14(10):1046. doi: 10.3390/pathogens14101046 (PMC12566916; doi:10.3390/pathogens14101046)

Figure S2. Seasonal–trend decomposition of the quarterly incidence rate of drug-resistant tuberculosis, Paraná, Brazil, 2012–2023.

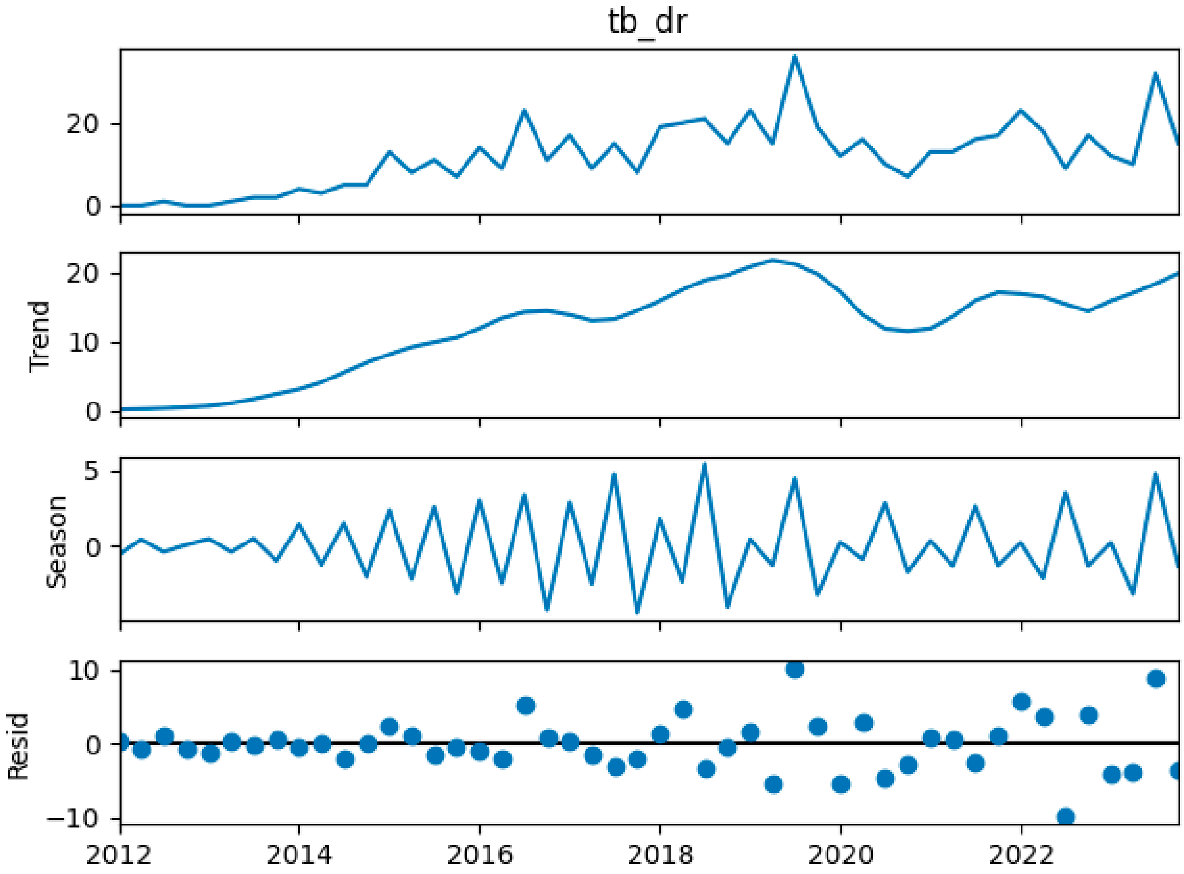

Supplement: Supplementary file 1 [file pathogens-14-01046-s001.zip › Figure S2.pdf]
