# Supplementary material for: From Patterns to Projections: A Spatiotemporal Distribution of Drug-Resistant Tuberculosis in Paraná, Brazil (2012–2023)
Source: Pathogens. 2025 Oct 16;14(10):1046. doi: 10.3390/pathogens14101046 (PMC12566916; doi:10.3390/pathogens14101046)
Supplement: Supplementary file 1 [file pathogens-14-01046-s001.zip › Table S1.pdf]

Supplementary material Table S1. Political-Administrative Health Division of Paraná State, Brazil

Health Macro-region	(ID) Health Region	Municipalities
East	(01) Paranaguá	Antonina, Guaraqueçaba, Guaratuba, Matinhos, Morretes, Paranaguá*, Pontal do Paraná (n = 7)
East	(02) Curitiba	Adrianópolis, Agudos do Sul, Almirante Tamandaré, Araucária, Balsa Nova, Bocaiúva do Sul, Campina Grande do Sul, Campo do Tenente, Campo Largo, Campo Magro, Cerro Azul, Colombo, Contenda, Curitiba*, Doutor Ulysses, Fazenda Rio Grande, Itaperuçu, Lapa, Mandirituba, Piên, Pinhais, Piraquara, Quatro Barras, Quitandinha, Rio Branco do Sul, Rio Negro, São José dos Pinhais, Tijucas do Sul, Tunas do Paraná (n = 29)
East	(03) Ponta Grossa	Arapoti, Carambeí, Castro, Ipiranga, Ivaí, Jaguariaíva, Palmeira, Piraí do Sul, Ponta Grossa*, Porto Amazonas, São João do Triunfo, Sengés (n = 12)
East	(04) Irati	Fernandes Pinheiro, Guamiranga, Imbituva, Inácio Martins, Irati*, Mallet, Rebouças, Rio Azul, Teixeira Soares (n = 9)
East	(05) Guarapuava	Boa Ventura de São Roque, Campina do Simão, Candói, Cantagalo, Foz do Jordão, Goioxim, Guarapuava*, Laranjal, Laranjeiras do Sul, Marquinho, Nova Laranjeiras, Palmital, Pinhão, Pitanga, Porto Barreiro, Prudentópolis, Reserva do Iguaçu, Rio Bonito do Iguaçu, Turvo , Virmond (n = 20)
East	(06) União da Vitória	Antônio Olinto, Bituruna, Cruz Machado, General Carneiro, Paula Freitas, Paulo Frontin, Porto Vitória, São Mateus do Sul, União da Vitória* (n = 9)
West	(07) Pato Branco	Bom Sucesso do Sul, Chopinzinho, Clevelândia, Coronel Domingos Soares, Coronel Vivida, Honório Serpa, Itapejara d'Oeste, Mangueirinha, Mariópolis, Palmas, Pato Branco*, São João, Saudade do Iguaçu, Sulina, Vitorino (n = 15)
West	(08) Francisco Beltrão	Ampére, Barracão, Bela Vista do Carobá, Boa Esperança do Iguaçu, Bom Jesus do Sul, Capanema, Cruzeiro do Iguaçu, Dois Vizinhos, Enéas Marques, Flor da Serra do Sul, Francisco Beltrão*, Manfrinópolis, Marmeleiro, Nova Esperança do Sudoeste, Nova Prata do Iguaçu, Pérola d'Oeste, Pinhal de São Bento, Planalto, Pranchita, Realeza, Renascença, Salgado Filho, Salto do Lontra, Santa Izabel do Oeste, Santo Antônio do Sudoeste, São Jorge d'Oeste, Verê (n = 27)
West	(09) Foz do Iguaçu	Foz do Iguaçu*, Itaipulândia, Matelândia, Medianeira, Missal, Ramilândia, Santa Terezinha de Itaipu, São Miguel do Iguaçu, Serranópolis do Iguaçu (n = 9)
West	(10) Cascavel	Anahy, Boa Vista da Aparecida, Braganey, Cafelândia, Campo Bonito, Capitão Leônidas Marques, Cascavel*, Catanduvas, Céu Azul, Corbélia, Diamante do Sul, Espigão Alto do Iguaçu, Formosa do Oeste, Guaraniaçu, Ibema, Iguatu, Iracema do Oeste, Jesuítas, Lindoeste, Nova Aurora, Quedas do Iguaçu, Santa Lúcia, Santa Tereza do Oeste, Três Barras do Paraná, Vera Cruz do Oeste (n = 25)

Northwest	(11) Campo Mourão	Altamira do Paraná, Araruna, Barbosa Ferraz, Boa Esperança, Campina da Lagoa, Campo Mourão*, Corumbataí do Sul, Engenheiro Beltrão, Farol, Fênix, Goioerê, Iretama, Janiópolis, Juranda, Luiziana, Mamborê, Moreira Sales, Nova Cantu, Peabiru, Quarto Centenário, Quinta do Sol, Rancho Alegre d'Oeste, Roncador, Terra Boa, Ubiratã (n = 25)
Northwest	(12) Umuarama	Alto Paraíso, Alto Piquiri, Altônia, Brasilândia do Sul, Cafezal do Sul, Cruzeiro do Oeste, Douradina, Esperança Nova, Francisco Alves, Icaraíma, Iporã, Ivaté, Maria Helena, Mariluz, Nova Olímpia, Perobal, Pérola, São Jorge do Patrocínio, Tapira, Umuarama*, Xambrê (n = 21).
Northwest	(13) Cianorte	Cianorte*, Cidade Gaúcha, Guaporema, Indianópolis, Japurá, Jussara, Rondon, São Manoel do Paraná, São Tomé, Tapejara, Tuneiras do Oeste (n = 10)
Northwest	(14) Paranavaí	Alto Paraná, Amaporã, Cruzeiro do Sul, Diamante do Norte, Guairaçá, Inajá, Itaúna do Sul, Jardim Olinda, Loanda, Marilena, Mirador, Nova Aliança do Ivaí, Nova Londrina, Paraíso do Norte, Paranapoema, Paranavaí*, Planaltina do Paraná, Porto Rico, Querência do Norte, Santa Cruz de Monte Castelo, Santa Isabel do Ivaí, Santa Mônica, Santo Antônio do Caiuá, São Carlos do Ivaí, São João do Caiuá, São Pedro do Paraná, Tamboara, Terra Rica (n = 28)
Northwest	(15) Maringá	Ângulo, Astorga, Atalaia, Colorado, Doutor Camargo, Floraí, Floresta, Flórida, Iguaraçu, Itaguajé, Itambé, Ivatuba, Lobato, Mandaguaçu, Mandaguari, Marialva, Maringá*, Munhoz de Melo, Nossa Senhora das Graças, Nova Esperança, Ourizona, Paiçandu, Paranacity, Presidente Castelo Branco, Santa Fé, Santa Inês, Santo Inácio, São Jorge do Ivaí, Sarandi, Uniflor (n = 30)
North	(16) Apucarana	Apucarana*, Arapongas, Bom Sucesso, Borrazópolis, Califórnia, Cambira, Faxinal, Grandes Rios, Jandaia do Sul, Kaloré, Marilândia do Sul, Marumbi, Mauá da Serra, Novo Itacolomi, Rio Bom, Sabáudia, São Pedro do Ivaí (n = 17)
North	(17) Londrina	Alvorada do Sul, Assaí, Bela Vista do Paraíso, Cafeara, Cambé, Centenário do Sul, Florestópolis, Guaraci, Ibiporã, Jaguapitã, Jataizinho, Londrina*, Lupionópolis, Miraselva, Pitangueiras, Porecatu, Prado Ferreira, Primeiro de Maio, Rolândia, Sertanópolis, Tamarana (n = 21)
North	(18) Cornélio Procópio	Abatiá, Andirá, Bandeirantes, Congonhinhas, Cornélio Procópio*, Itambaracá, Leópolis, Nova América da Colina, Nova Fátima, Nova Santa Bárbara, Rancho Alegre, Ribeirão do Pinhal, Santa Amélia, Santa Cecília do Pavão, Santa Mariana, Santo Antônio do Paraíso, São Jerônimo da Serra, São Sebastião da Amoreira, Sapopema, Sertaneja, Uraí (n = 21)
North	(19) Jacarezinho	Barra do Jacaré, Cambará, Carlópolis, Conselheiro Mairinck, Figueira, Guapirama, Ibaiti, Jaboti, Jacarezinho*, Japira, Joaquim Távora, Jundiaí do Sul, Pinhalão, Quatiguá, Ribeirão Claro, Salto do Itararé, Santana do Itararé, Santo Antônio da Platina, São José da Boa Vista, Siqueira Campos, Tomazina, Wenceslau Braz (n = 22)

West	(20) Toledo	Assis Chateaubriand, Diamante d'Oeste, Entre Rios do Oeste, Guaíra, Marechal Cândido Rondon, Maripá, Mercedes, Nova Santa Rosa, Ouro Verde do Oeste, Palotina, Pato Bragado, Quatro Pontes, Santa Helena, São José das Palmeiras, São Pedro do Iguaçu, Terra Roxa, Toledo*, Tupãssi (n = 18)
East	(21) Telêmaco Borba	Curiúva, Imbaú, Ortigueira, Reserva, Telêmaco Borba*, Tibagi, Ventania (n = 7)
North	(22) Ivaiporã	Arapuã, Ariranha do Ivaí, Cândido de Abreu, Cruzmaltina, Godoy Moreira, Ivaiporã*, Jardim Alegre, Lidianópolis, Lunardelli, Manoel Ribas, Mato Rico, Nova Tebas, Rio Branco do Ivaí, Rosário do Ivaí, Santa Maria do Oeste, São João do Ivaí (n = 16)
(n = 4)	(n = 22)	(n = 399)

Note. ID:identification number of the Health Region; Asterisks (*) Seat municipality of the Health Region.
