# Supplementary material for: From Patterns to Projections: A Spatiotemporal Distribution of Drug-Resistant Tuberculosis in Paraná, Brazil (2012–2023)
Source: Pathogens. 2025 Oct 16;14(10):1046. doi: 10.3390/pathogens14101046 (PMC12566916; doi:10.3390/pathogens14101046)
Supplement: Supplementary file 1 [file pathogens-14-01046-s001.zip › Table S2.pdf]

Supplementary material Table S2. Data sources, definitions, and links for indicators included in the analysis

Dimension	Indicator	Definition	Source	Link	Year
Morbidity	Reported tuberculosis cases during the study period, Paraná, Brazil	Number of TB cases notified in the study period	Notifiable Diseases Information System (SINAN) – DATASUS	https://datasus.saude.gov.br/transferencia-de-arquivos/	2025
Demographic	Resident population by municipality, Paraná, Brazil	Total population residing in each municipality according to census for rate calculation (descriptive and spatial analysis)	Brazilian Institute of Geography and Statistics (IBGE) – 2022 Census	https://www.ibge.gov.br/	2022
Demographic	Population projections by sex, ethnicity and age group per municipality, Paraná, Brazil	Population distribution by sex, ethnicity and age group in each municipality based on census data (descriptive analysis)	Brazilian Institute of Geography and Statistics (IBGE) – 2010 Census<br><br>Tabulated by Institute for Economic and Social Development of Paraná (IPARDES)	https://www.ipardes.pr.gov.br/Pagina/Tabelas-Censos-Demograficos	2010
Demographic	Population projections by sex, ethnicity and age group per municipality, Paraná, Brazil	Population distribution by sex, ethnicity and age group in each municipality based on census data (descriptive analysis)	Brazilian Institute of Geography and Statistics (IBGE) – 2022 Census<br><br>Tabulated by Institute for Economic and Social Development of Paraná (IPARDES)	https://www.ipardes.pr.gov.br/Pagina/Tabelas-Censos-Demograficos	2022
Notification	Notification form (TB)	Standardized form used nationwide, completed by healthcare professionals to notify suspected or confirmed TB cases in SINAN	Sinan NET Tuberculosis	https://portalsinan.saude.gov.br/images/documentos/Agravos/Tuberculose/Tuberculose_v5.pdf	2014
Metadata	SINAN Data Dictionary	It is used nationwide to ensure consistent data collection and interpretation across health services	Data Dictionary – SINAN v5.0 Tuberculosis	https://portalsinan.saude.gov.br/images/documentos/Agravos/Tuberculose/DICI_DADOS_Tuberculose.pdf	2020
Geographic	Shapefiles of municipalities and Health Regions, Paraná, Brazil	Official cartographic base used to generate thematic maps and spatial analysis	Brazilian Institute of Geography and Statistics (IBGE) – Cartographic Base	https://www.ibge.gov.br/geociencias/cartas-e-mapas/bases-cartograficas-continuas/15759-brasil.html	2023
