# Supplementary material for: From Patterns to Projections: A Spatiotemporal Distribution of Drug-Resistant Tuberculosis in Paraná, Brazil (2012–2023)
Source: Pathogens. 2025 Oct 16;14(10):1046. doi: 10.3390/pathogens14101046 (PMC12566916; doi:10.3390/pathogens14101046)
Supplement: Supplementary file 1 [file pathogens-14-01046-s001.zip › Table S3.pdf]

Supplementary material Table S3. Diagnostic algorithm for tuberculosis according to the Manual of the Brazilian Ministry of Health.

Laboratory surveillance is a key component of tuberculosis (TB) epidemiological surveillance. It encompasses a set of coordinated actions and mechanisms aimed at ensuring accurate and timely diagnosis, as well as monitoring treatment outcomes and the effectiveness of TB control strategies.

This diagnostic algorithm follows the recommendations established in the Tuberculosis Control Manual published by the Ministry of Health of Brazil, and is uniformly applied across laboratories in the national public diagnostic network.

Step	Decision Point	Action
1	Person with suspected tuberculosis	Perform Rapid Molecular Test for Tuberculosis (RMT-TB)
2A	Result: "*Mycobacterium tuberculosis* detected"	Considered a tuberculosis case
3A.1	Rifampicin resistance detected	Repeat RMT-TB (with a new sample) + perform culture + drug susceptibility testing<br>Immediately refer to a tertiary healthcare facility
3A.2	Rifampicin resistance not detected	Perform culture and drug susceptibility testing<br>Start treatment with the basic tuberculosis regimen (first-line drugs)
2B	Result: "*Mycobacterium tuberculosis* not detected"	Assess for ongoing symptoms
3B.1	No symptoms	Rule out tuberculosis diagnosis
3B.2	Symptoms persist	Continue investigation with culture and drug susceptibility testing

1. New tuberculosis cases may occur in the general population, healthcare professionals, incarcerated individuals, homeless people, Indigenous peoples, and individuals who are contacts of persons with drug-resistant tuberculosis.
2. The RMT-TB Xpert® MTB/RIF Ultra cartridge.
3. A result of "*Mycobacterium tuberculosis* detected" is valid except when trace amounts are found. For this specific population, a "trace" result is not confirmatory for tuberculosis, and further investigation should continue based on clinical evaluation.
4. Drug susceptibility testing should be performed using either solid or liquid culture-based methods.
5. Tertiary referral refers to a specialized outpatient clinic for the treatment of drug-resistant tuberculosis. The individual must be referred immediately. In this facility, a medical evaluation and the appropriate clinical management must be conducted within seven days. The result of the culture and drug susceptibility test must be sent to the tertiary facility.
6. Begin treatment with the basic tuberculosis regimen, and reassess treatment once the results of the culture and drug susceptibility test are available.

Reference

19. Brazil. Ministry of Health. Guidelines: Good Laboratory Practices for Tuberculosis and Other Mycobacteria in Brazil. Brasília: Ministry of Health; 2022. 492 p.
