# Supplementary figures and images for: From Patterns to Projections: A Spatiotemporal Distribution of Drug-Resistant Tuberculosis in Paraná, Brazil (2012–2023)
Source: Pathogens. 2025 Oct 16;14(10):1046. doi: 10.3390/pathogens14101046 (PMC12566916; doi:10.3390/pathogens14101046)

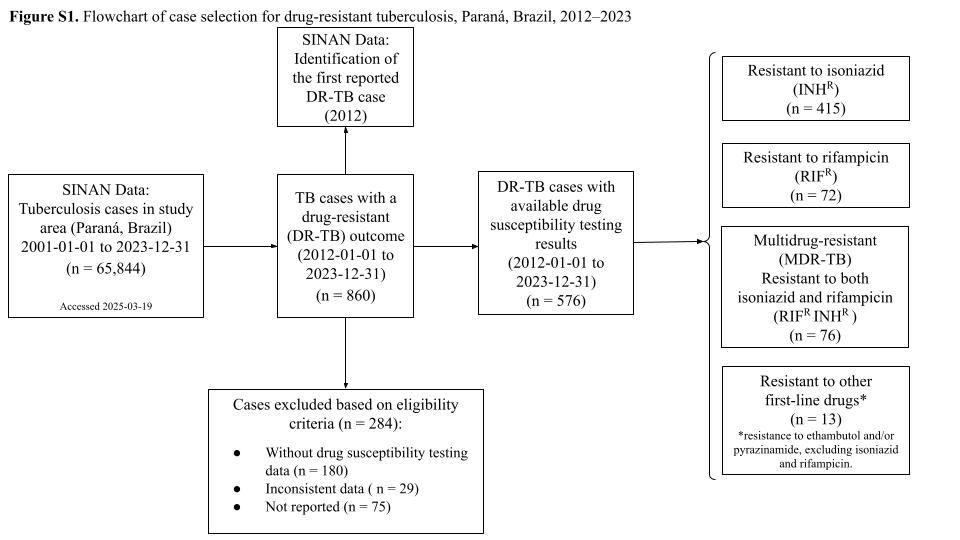

Supplement: Supplementary file 1 [file pathogens-14-01046-s001.zip › FigureS1.jpg]
